# Determinants of the Lifestyle Changes during COVID-19 Pandemic in the Residents of Northern Italy

**DOI:** 10.3390/ijerph17176287

**Published:** 2020-08-28

**Authors:** Raffaella Cancello, Davide Soranna, Gaia Zambra, Antonella Zambon, Cecilia Invitti

**Affiliations:** 1 Obesity Unit—Laboratory of Nutrition and Obesity Research, Department of Endocrine and Metabolic Diseases, IRCCS Istituto Auxologico Italiano, 20145 Milan, Italy; 2 Istituto Auxologico Italiano, IRCCS, Biostatistic Unit, 20145 Milan, Italy; d.soranna@auxologico.it (D.S.); g.zambra@auxologico.it (G.Z.); antonella.zambon@unimib.it (A.Z.); 3 Department of Statistics and Quantitative Methods, University of Milano-Bicocca, 20126 Milan, Italy; 4 Research laboratory of Preventive Medicine, IRCCS Istituto Auxologico Italiano, 20122 Milan, Italy; invitti@auxologico.it

**Keywords:** COVID-19, lockdown, lifestyle, food habits, diet, physical activity, sleep, smoking, survey, health

## Abstract

Background: The confinement recommended during COVID-19 pandemic could affect behavior and health. Methods: We conducted a self-reported survey in northern Italy to observe the lockdown effects on lifestyle changes and to assess their determinants. Prevalence Odds Ratio and Prevalence Risk Ratio were determined. Results: 490 adults (84% female) completed the survey: 13% and 43% reported improved and unchanged sleep quality, respectively, while 43% had insomnia symptoms. Among the 272 active subjects in pre-lockdown, 14% continued habitual exercising, 18% increased it and 68% reduced it; 27% of sedentary subjects started physical exercise; 34% reported an improvement in diet quality; 42% increased food intake and 13% decreased it; and 38% of the smokers increased cigarette consumption. Age and the pre-lockdown habit of regular physical exercising were the mainly determinants of lifestyle changes whereas BMI, gender, and the presence of chronic diseases did not. Living with other people increased the likelihood of increasing the food intake (*p* = 0.002). Conclusions: More than a third of people were able to positively reorganize their lives during the forced home confinement. It is worth to disseminate information to preserve a healthy lifestyle even when confined at home.

## 1. Introduction

In December 2019, novel coronavirus (2019-nCoV)–infected pneumonia occurred in Wuhan, China [1]. The number of cases increased rapidly and on 11 March 2020, the World Health Organization declared a COVID-19 pandemic [2]. As of 6 June 2020, COVID-19 counted nearly 7 million cases worldwide with nearly 400,000 deaths. Italy, with over 33,000 deaths, was certainly one of the most affected countries, particularly in the northern regions that accounted for the 85% of cases [3,4]. As of 15 April 2020, the Lombardy region accounted for 37% of cases and 53% of deaths of the country [5]. From 10 March to 4 May, the Italian Government implemented several restrictive measures to contain the infection spread [6]. The containment measures limited people leave the house only for urgent needs only such as shopping for foods and serious health reasons and most working subjects converted the habitual occupation into “at home” smart working. Data from an American company that develops wearable devices that track an individual’s physical activity level, indicate that during confinement for COVID-19, the physical activity underwent a substantial decrease all over the world [7]. Similar results were observed in young physically active subjects in Sicily, Italy [8]. Changes in routine, uncertainty, stress, social isolation, concerns about the situation and health as well as the reduced exposure to light can worsen sleep quality [9] and generate behaviors such as worsening of eating habits and sedentary lifestyle that could reciprocally influence each other seriously affecting health outcomes and quality of life. On the contrary, the greater availability of time to spend with the family, the greater interaction via social media and for those with outdoor spaces, the greater exposure to light could improve the quality of life and consequently sleep. Similarly, eating habits could improve due to the greater availability of time to cook healthily. Indeed, a survey conducted in Spain evidenced that prevalence of health risk behaviors decreased during the confinement due to COVID-19 [10] except for screen exposure. An Italian survey on lifestyle habits during COVID-19 pandemic showed that more than one third of subjects feel to have improved their lifestyle habits [11]. In these situations, the maintaining of a healthy lifestyle is very important to avoid adopting habits that facilitate the onset of risk factors for chronic non-communicable diseases (such as diabetes, cardiovascular disease, obesity, and cancer) difficult to eradicate after the end of the emergency.

We conducted a self-reported survey among the residents of northern Italy, which was the Italian area most affected by COVID-19 pandemic, from 15 April to 4 May 2020, to observe the effects of the lockdown on the reported lifestyle habits changes. The purpose of this survey was to give a picture of the Italian population perception of changes occurred in the main components of lifestyle (i.e., eating habits, physical activity, sleep, and smoking) and (based on the reported answers) to identify their determinants.

## 2. Materials and Methods

### 2.1. Survey

We posted the survey on social media platforms (IRCCS Istituto Auxologico Italiano newsletter, which is mainly addressed to the people attending the structures in Lombardy and in Piedmont of the Institute and is shared on Facebook, and Instagram platform) to collect self-reported data on lifestyle changes during lockdown. The survey was addressed to the “digital user” adults from 15 April to 4 May 2020 (day of restrictions suspension). Participants independently completed an anonymous online questionnaire, explicitly agreeing to participate in the survey. The study protocol was approved by the Ethics Committee of IRCCS-Istituto Auxologico Italiano (approval code number #2020051905).

### 2.2. Questionnaire

The questionnaire was built by using Google Form, a free tool that allows collecting information through a survey or a personalized quiz (the full version of the questionnaire is available in the Supplementary Materials). The information was automatically linked to an Excel spreadsheet that at the end contained the independently compiled answers that users have given. The questionnaire included 31 questions (multiple choice, single choice, numeric, and open ended) on baseline data (age, gender, zip code, education, current work conditions, number of cohabitants, size of the house, presence of chronic diseases, occurrence of COVID-19 infection among relatives/friends/himself, smoking habits, weekly hours of physical activity, weight, and height) and changes during lockdown (weight, sleep quality, physical activity, cigarette consumption, appetite, food purchases and intake, quality of the diet, and use of supplements). The zip code was used to give the sample a geographical location in Italian country.

### 2.3. Statistical Analysis

Continuous variables are shown as median and interquartile range (IQR). Categorical data are reported as frequencies and proportions. The determinants of changes for the three main lifestyle components (i.e., diet/eating habits, physical activity, and sleep) were established considering the outcome “change” at 3 levels: improve/increase, worsening/decrease, and no change of the variable. All the association estimates are reported as prevalence odds ratio (POR) and relative 95% confidence interval (95% CI). Moreover, for the lifestyle component “smoking” we performed the analysis only in subjects who declare themselves smokers in the pre-lockdown period. In this case a multivariable Poisson regression with robust variance [12] was applied to estimate the association between the determinants and increase in cigarette consumption. These association estimates are reported as prevalence risk ratio (PRR) and relative 95% CI. All analyses were performed using the Statistical Analysis System Software (version 9.4; SAS Institute, Cary, NC, USA). Statistical significance was set at the 0.05 level. All *p* values were 2-sided.

## 3. Results

### 3.1. Socio-Demographic Characteristics of Survey Responders

The survey was self-completed by 497 responders almost exclusively (90%) resident in Lombardy and Piedmont (northern Italy). Questionnaires of seven responders were eliminated from the analysis because they were completed by subjects aged less than 18 years. The characteristics of the 490 adult responders according the age group are reported in Table 1. The sample was mostly composed by females (84%) and subjects with a high educational level (58%); 35% of the employed responders converted the habitual occupation in smart working and 23% suspended the work. The proportion of overweight and obese subjects was 19% and 13%, respectively. Most subjects (86%) cohabited with other persons. Fifty four percent of the responders had a relative or a friend infected by Covid-19; five subjects had personally contracted the viral infection (three female and two men, four normal weight and one obese, two with chronic diseases, and all non-smokers). More than half of responders were physically active.

### 3.2. Lifestyle Changes during Lockdown

#### Sleep Quality

**Figure 1:**
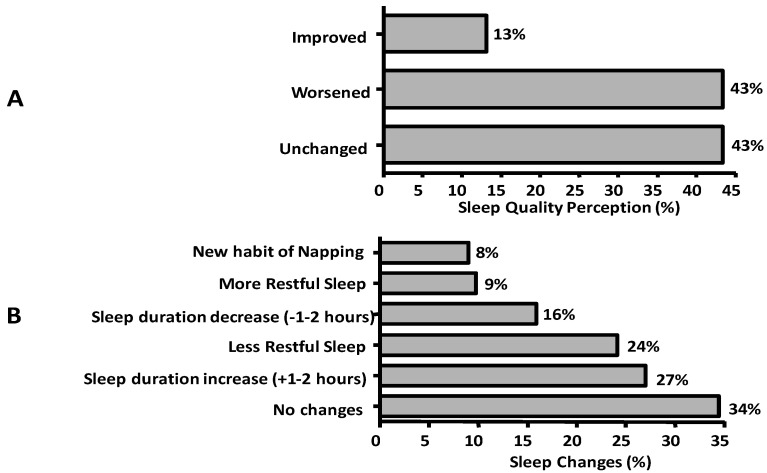
Frequencies of perceived quality of sleep (**A**) and of changes in sleep duration and feeling of restful sleep (**B**).

During the lockdown, 13% and 43% of responders reported improved and unchanged the sleep quality, respectively; 43% reported symptoms of insomnia and 4% a new-onset persistent insomnia (Figure 1A). The sleep duration increased in 22% and decreased in 13% of the responders; 8% of the subjects defined the sleep more restful and 20% less restful (Figure 1B).

### 3.3. Physical Activity

**Figure 2:**
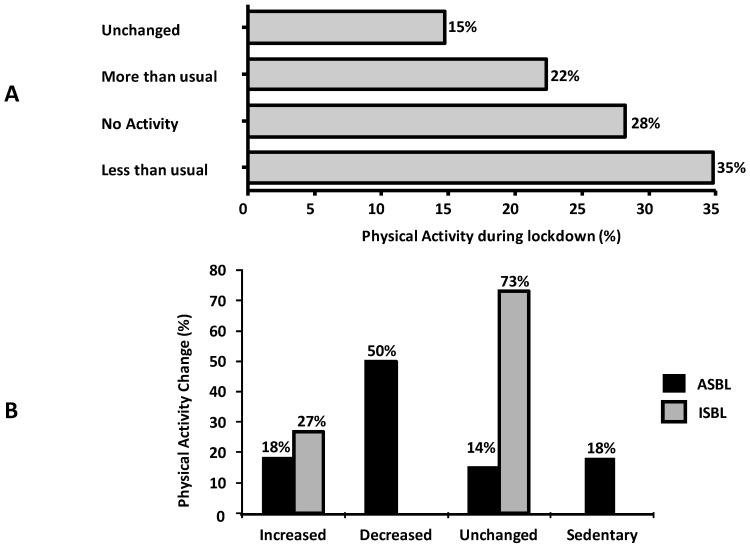
Frequencies of the reported changes in physical activity (**A**); physical activity changes in subjects who were active before lockdown (Active Subjects Before Lockdown (ASBL), black columns) and in subjects who were inactive before lockdown (Inactive Subjects Before Lockdown (ISB), grey columns) (**B**).

During lockdown 22% of the whole sample reported to perform physical activity more than usual (Figure 2A). Among the 272 subjects who performed more than 2 h of physical activity “per week” before the lockdown (defined as “active” since performing ≥150 min of exercise per week), 14% continued the habitual activity, 50% reduced the hours of physical activity, 18% became inactive, and 18% increased it. On the contrary, 27% of the 218 subjects who were sedentary before the implementation of restrictive measures (inactive) started practicing physical exercise (Figure 2B).

### 3.4. Food Habits and Nutrition

**Figure 3:**
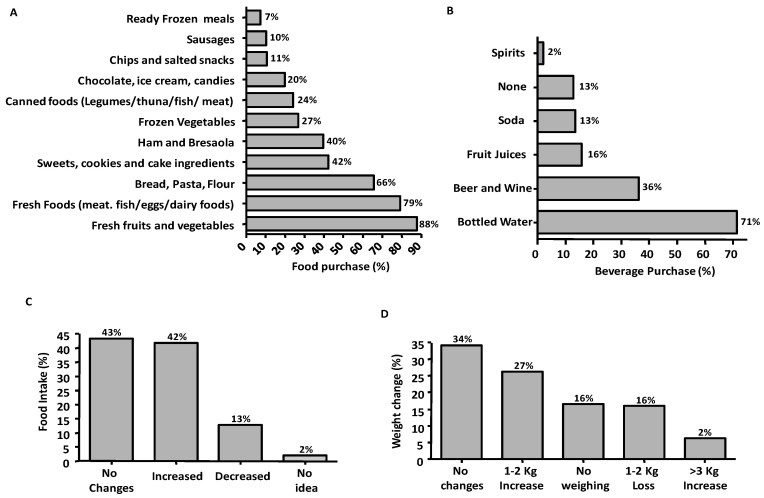
Frequencies of the more frequently chosen foods (**A**) and beverages (**B**) during lockdown. Changes in food intake (**C**) and weight (**D**) during lockdown.

During lockdown, 70% of subjects planned the food supply once a week and almost all prepared the list of foods to buy (91%). Figure 3A reports the frequency of the most purchased food categories during lockdown. The responders reported that they bought more fresh foods (fruits, vegetables, meat, and fish) followed by bread, pasta, flour, and sweets/ingredients for the homemade cakes preparation and less than a quarter purchased beer and wine (Figure 3B). During the lockdown, 34% and 19% perceived to have improved and worsened respectively the quality of the diet; 42% and 13% of subjects perceived to have increased and decreased the food consumption (Figure 3C). The subjects who had increased food consumption compared to those reporting no changes, consumed more snacks/appetizers (41.5% vs. 19.8%) and reported as main food choice determinant the “need to keep costs down” (40% vs. 6%) followed by the “cooking to spend time” (19% vs. 6%). Most of the 63 responders who declared to have decreased the food intake were normal-weight and active before lockdown (62%) and reported a loss of appetite (56%). Among the 408/490 people who weighed themselves during lockdown, 42% reported a stable weight, 39% gained weight (79% of whom increased the food intake) and 19% lost 1–2 kg of weight (Figure 3D).

Twenty three percent of subjects reported to have started taking supplements (vitamins and/or minerals) during lockdown with the intent of strengthening the immune system.

### 3.5. Smoking Habits

Among the 105 habitual smokers, 38% reported an increase in the cigarette consumption that was associated with an increase in food intake in 60% of them.

### 3.6. Determinants of Lifestyle Changes

Table 2, Table 3 and Table 4 show the results of multinomial logistic regressions to identify the determinants of change in the main components of lifestyle (sleep, physical activity, and food intake respectively) during lockdown. From Table 2 it appears that none of the considered determinants was associated to the change in the sleep quality except for the age since subjects older than 60 years had a reduced probability of worsening the sleep compared to younger people. Concerning the changes in physical activity, Table 3 shows that individuals older than 30 years and those who reduced their food intake, were less and more likely to increase physical activity respectively. Higher educational levels were associated to a lower probability to decrease the physical activity, whereas having a relative/friend or himself with Covid-19 increased the risk of changing physical activity in both directions (increase or reduction). Regarding the changes in food intake, subjects who cohabited with other persons had a higher risk of increasing food consumption, whereas those who increased the physical activity were three times more likely to also reduce food intake (Table 4). The increase in the cigarette consumption was more likely in subjects who increased the food intake and underwent a change in the sleep quality (either improved or worsened) (Table 5).

## 4. Discussion

We conducted a self-reported survey in northern Italy to estimate the COVID-19 lockdown effects on lifestyle and to assess their determinants during the time of restrictive measures to contain the infection spread. The survey showed that more than one third of the residents of northern Italy were able to positively reorganize their habits in the face of this sudden and unexpected situation. Indeed, 56% of people reported that they had not changed their sleep or even improved it, 32% of the subjects who were active before the lockdown continued the habitual exercise or increased it, 27% of the subjects who were sedentary began to exercise, and 34% of the responders had the impression that they had improved the quality of the diet. An equally large group of individuals seems not to have found the strength to accommodate the lockdown and become sedentary and/or worsened sleep quality and increased food and cigarette consumption. Finally, a minority of subjects likely reacted in an “exaggerated” unhealthy way, by increasing physical activity, becoming unappetizing and losing weight. When we tried to identify the factors that determined the direction of change in lifestyle during lockdown, we observed that the age and the pre-lockdown habit of physical exercising were the mainly determinants of lifestyle changes whereas BMI, gender, and the presence of chronic diseases did not. In detail, the older age (>60 years) seems to have protected from the deterioration of the sleep quality suggesting that younger people who had a greater impact on social interactions and employment likely suffered greater anxiety, which resulted in a worse sleep. These observations were never reported before and could be relevant for the development of population advice for maintaining health if it could be necessary to adopt new restrictive measures to contain the pandemic in the future.

In line with our results, a study of Huang and Zhao reported that being younger than 35 years and following COVID-19 news for more than 3 h a day was associated with elevated levels of anxiety compared with those who were older and followed the updates the least [13].

A survey conducted in residents mainly from central and southern Italy reported that during lockdown, people slept more hours than before [11] and similarly in Spain the number of short sleepers decreased during the first three weeks of confinement [10]. In our survey, 22% of subjects reported sleeping 1–2 h more than usual, this result however does not necessarily reflect an improvement in the quality of sleep because 43% of subjects described symptoms of insomnia and 20% perceived their sleep as less restful.

About a third of the responders to our survey remained active during lockdown suggesting that the promotion of exercise as a health-promoting tool is having results. The desire to remain active is also demonstrated by the parallel worldwide growth in the search for the term “home training” and “COVID-19” in Google [14]. In our cohort, the most receptive people seemed to be those with a higher educational level who were less likely to reduce physical activity and younger people because being older than 30 years made the increase in physical activity during home confinement less likely. However a recent study showed that the metabolic control of Type 1 Diabetes in adolescents did not worsen during the restrictions due to COVID-19 pandemics and further improved in those who continued physical activity during the quarantine [15], demonstrating the power of regular physical activity as an essential strategy for healthy living during the COVID-19 crisis, especially for individuals with chronic diseases. In the present study, only 23% of subjects reported to be affected by a chronic disease and the questionnaire did not investigate the type of the chronic disease. It was therefore impossible to deepen the role of single or combined pathologies of on lifestyle changes. Further research will be needed to investigate this topic.

The survey revealed a strong association between the behavior of increasing physical activity and reducing food consumption. As the majority of people who reduced the food intake was normal-weight, it may assumed that the behavior of subjects who simultaneously increased physical activity and reduced nutrition was generated by a loss of appetite (possibly related to anxiety), rather than driven by the willingness to achieve a healthy weight.

As the opposite, the cohabitation with other persons was the main determinant of an increase in food intake which was accompanied by a greater consumption of snacks and appetizers in addition to the three main meals. It is difficult to interpret this behavior which can be attributed to the need for consolatory/conviviality or to the availability of time. The positive finding is however that the purchases of food in the lockdown period were mainly oriented towards healthy food and drinks, in agreement with the good adhesion to the Mediterranean diet recorded by Di Renzo et al. during lockdown [11].

Finally, our survey shows that more than a third of smokers increased the cigarette consumption during home confinement and this conduct was associated with an increase in food intake and changes (in both directions) of sleep quality. This finding further support the hypothesis of a state of psychological distress that we have not investigated in this survey.

The strength of the present study is the inclusion of multiple health behaviors, and the timing of data collection relative to lockdown restrictions in Italy.

This study has the following limitations: firstly, we cannot exclude a bias due to the self-reported responses; secondly, we did not investigate the association between lifestyle and psychological distress, and thirdly, we cannot exclude a gender-related effect that the small number of men in our responders group may have hidden.

Encouraging the maintenance of healthy food choices, regular mealtime, the practice of physical activity at home should be the way to make the population aware of the need for healthy state preservation. The promotion of correct lifestyles is important for the protection of health, but it becomes even more so in case of forced confinement at home.

## 5. Conclusions

In sudden and unexpected critical situations, such as the current COVID-19 pandemic, more than a third of people resident in northern Italy demonstrate that they are able to positively reorganize or maintain their lifestyle. Encouraging the maintenance of healthy food choices, regular mealtime, the practice of physical activity at home should be the way to make the population aware of the need for healthy state preservation. The promotion of correct lifestyles is important for the protection of health, but it becomes even more so in case of forced confinement at home.

It is worth to provide suggestions on the way to maintain a correct lifestyle through video- or app-based supports but also by non-digital channels (such as TV, newspapers, journals, posters, and leaflets) in order to reach also less technological people.

## Figures and Tables

**Table 1:** Characteristics of the 490 subjects who participated in the survey.

	Overall
	*N* = 490
Women *N (%)*:	410 (83.67%)
Age, *N (%)*:	
≤30 years	71 (14.5%)
31–60 years	319 (65.1%)
>60 years	100 (20.4%)
BMI, kg/m^2^ *median [IQR]*	22.89 [20.39–26.03]
Smokers *N (%)*:	105 (21.4%)
Chronic diseases, *N (%)*:	114 (23.3%)
Educational level, *N (%)*:	
No University	204 (41.6%)
University	286 (58.4%)
Work situation, *N(%)*:	
Usual workplace	62 (12.6%)
Unemployed	135 (27.5%)
Smart-working	172 (35.1%)
Suspension of work	115 (23.5%)
House size, *N (%):*	
Less than 100 m^2^	267 (54.60%)
More than 100 m^2^	222 (45.40%)
Cohabitation *N(%)*:	
alone	68 (13.8%)
not alone	422 (86.12%)
Relatives/himself with COVID-19, *N(%)*:	
No	225 (45.91%)
Yes	265 (54.19%)
Habitual physical activity levels, *N(%):*	
Sedentary	130 (26.5%)
1 h weekly	88 (18%)
2–3 h weekly	164 (33.5%)
>4 h weekly	108 (22.0%)

IQR = Interquartile range.

**Table 2:** Prevalence Odds Ratio (POR) for changes in the sleep quality.

	Comparison Better vs. No Change		Comparison Worst vs. No Change	
	POR (95% CI)	*p*-Value	POR (95% CI)	*p*-Value
Age				
≤30 years	Ref.		Ref.	
31–60 years	0.78 (0.33; 1.87)	0.5782	0.90 (0.47; 1.72)	0.7427
>60 years	0.59 (0.19; 1.80)	0.3538	0.36 (0.16; 0.80)	0.0119
Gender				
Female	Ref.		Ref.	
Male	1.08 (0.50; 2.34)	0.8363	0.68 (0.38; 1.19)	0.1759
BMI (kg/m^2^)	0.99 (0.94; 1.04)	0.6600	1.01 (0.99; 1.03)	0.3514
Smoking				
No	Ref.		Ref.	
Yes	1.31 (0.65; 2.65)	0.4556	1.43 (0.87; 2.35)	0.1639
Chronic diseases				
No	Ref.		Ref.	
Yes	0.73 (0.32; 1.67)	0.4548	1.01 (0.60; 1.69)	0.9714
Educational level				
No University	Ref.		Ref.	
University	1.33 (0.70; 2.52)	0.3768	0.84 (0.55; 1.29)	0.4324
Work situation				
Usual workplace	Ref.		Ref.	
Unemployed	0.98 (0.32; 3.06)	0.9777	1.29 (0.61; 2.70)	0.5039
Smart Working	1.54 (0.59; 4.04)	0.3768	1.12 (0.58; 2.16)	0.7260
Suspension	1.22 (0.43; 3.46)	0.7093	1.09 (0.54; 2.20)	0.8013
House size				
Less than 100 m^2^	Ref.		Ref.	
More than 100 m^2^	0.63 (0.34; 1.16)	0.1354	0.68 (0.44; 1.03)	0.0712
Cohabitation				
alone	Ref.		Ref.	
not alone	1.17 (0.49; 2.80)	0.7299	0.89 (0.48; 1.65)	0.7119
Relatives/himself with COVID-19				
No	Ref.		Ref.	
Yes	1.00 (0.56; 1.79)	0.9929	1.30 (0.86; 1.97)	0.2106
Food				
No change	Ref.		Ref.	
Decreased	1.82 (0.79; 4.19)	0.1592	1.30 (0.68; 2.48)	0.4298
Increased	1.09 (0.58; 2.07)	0.7895	1.44 (0.93; 2.24)	0.0990
Physical activity *N(%):*				
No change	Ref.		Ref.	
Decreased/no activity	0.85 (0.37; 1.94)	0.6933	1.46 (0.79; 2.71)	0.2278
Increased	1.55 (0.63; 3.79)	0.3383	1.39 (0.68; 2.84)	0.3642

**Table 3:** Prevalence Odds Ratio (POR) for changes in physical activity.

	Comparison Decreased/no Activity vs. No Change		Comparison Increased vs. No Change	
	POR (95% CI)	*p*-Value	POR (95% CI)	*p*-Value
Age				
≤30 years	Ref.		Ref.	
31–60 years	1.53 (0.62; 3.79)	0.3596	0.39 (0.15; 0.97)	0.0424
>60 years	1.22 (0.42; 3.55)	0.7208	0.21 (0.06; 0.71)	0.0123
Gender				
Female	Ref.		Ref.	
Male	1.18 (0.56; 2.49)	0.6616	0.48 (0.18; 1.32)	0.1545
BMI (kg/m^2^)	1.00 (0.98; 1.02)	0.8984	0.98 (0.93; 1.03)	0.3453
Smoking				
No	Ref.		Ref.	
Yes	1.00 (0.50; 1.98)	0.9985	0.78 (0.35; 1.72)	0.5338
Chronic diseases				
No	Ref.		Ref.	
Yes	2.02 (0.91; 4.52)	0.0849	1.58 (0.61; 4.08)	0.3455
Educational level				
No Univesrity	Ref.		Ref.	
University	0.36 (0.20; 0.67)	0.0013	0.65 (0.31; 1.33)	0.2334
Work situation				
Usual workplace	Ref.		Ref.	
Unemployed	0.52 (0.17; 1.58)	0.2464	0.97 (0.25; 3.69)	0.9588
Smart Working	0.58 (0.22; 1.57)	0.2849	1.43 (0.42; 4.86)	0.5681
Suspension	0.35 (0.13; 0.99)	0.0473	1.18 (0.34; 4.12)	0.7917
House size				
Less than 100 m^2^	Ref.		Ref.	
More than 100 m^2^	0.78 (0.44; 1.42)	0.4209	0.91 (0.46; 1.78)	0.7733
Cohabitation				
alone	Ref.		Ref.	
not alone	0.57 (0.23; 1.44)	0.2347	0.73 (0.25; 2.15)	0.5740
Relatives/himself with with COVID-19				
No	Ref.		Ref.	
Yes	2.49 (1.40; 4.42)	0.0019	2.53 (1.31; 4.86)	0.0055
Food				
No change	Ref.		Ref.	
Decreased	1.68 (0.59; 4.75)	0.3282	3.21 (1.06; 9.75)	0.0398
Increased	1.14 (0.63; 2.07)	0.6600	0.95 (0.48; 1.90)	0.8883
Sleep quality				
No change	Ref.		Ref.	
Improved	0.84 (0.36; 1.94)	0.6773	1.52 (0.61; 3.78)	0.3644
Worsened	1.44 (0.77; 2.68)	0.2509	1.43 (0.69; 2.94)	0.3321

**Table 4:** Prevalence Odds Ratio (POR) for changes in food intake.

	Comparison Decreased vs. No Change		Comparison Increased vs. No Change	
	POR (95% CI)	*p*-Value	POR (95% CI)	*p*-Value
Age				
≤30 years.	Ref.		Ref.	
31–60 years	1.18 (0.42; 3.27)	0.7560	0.56 (0.30; 1.06)	0.0744
>60 years	1.66 (0.53; 5.20)	0.3883	0.54 (0.25; 1.19)	0.1251
Gender				
Female	Ref.		Ref.	
Male	1.09 (0.50; 2.38)	0.8298	0.84 (0.48; 1.47)	0.5387
BMI (Kg/m^2^)	1.00 (0.97; 1.03)	0.9918	1.00 (0.98; 1.02)	0.8305
Smoking				
No	Ref.		Ref.	
Yes	0.97 (0.47; 1.99)	0.9241	1.03 (0.63; 1.69)	0.9007
Chronic diseases				
No	Ref.		Ref.	
Yes	1.16 (0.57; 2.36)	0.6729	0.76 (0.45; 1.29)	0.3077
Educational level				
No University	Ref.		Ref.	
University	1.09 (0.58; 2.05)	0.7786	0.90 (0.59; 1.38)	0.6348
Work situation				
Usual workplace	Ref.		Ref.	
Unemployed	0.75 (0.28; 2.02)	0.5731	0.92 (0.43; 2.00)	0.8421
Smart Working	0.66 (0.27; 1.60)	0.3596	1.76 (0.90; 3.46)	0.1003
Suspension	0.55 (0.20; 1.52)	0.2501	1.95 (0.96; 3.98)	0.0661
House size				
Less than 100 m^2^	Ref.		Ref.	
More than 100 m^2^	0.93 (0.50; 1.76)	0.8333	1.03 (0.67; 1.58)	0.8867
Cohabitation				
alone	Ref.		Ref.	
not alone	0.49 (0.24; 1.00)	0.0495	2.23 (1.13; 4.39)	0.0205
Relatives/himself with COVID-19				
No	Ref.		Ref.	
Yes	0.96 (0.53; 1.74)	0.8826	1.05 (0.7; 1.59)	0.8057
Sleep quality				
No change	Ref.		Ref.	
Improved	1.72 (0.75; 3.99)	0.2028	1.07 (0.56; 2.04)	0.8271
Worsened	1.27 (0.66; 2.44)	0.4741	1.44 (0.93; 2.23)	0.1007
Physical activity				
No change	Ref.		Ref.	
Decreased	1.81 (0.63; 5.15)	0.2679	1.18 (0.66; 2.13)	0.5780
Increased	3.42 (1.1; 10.64)	0.0338	0.98 (0.50; 1.93)	0.9509

**Table 5:** Prevalence Risk ratio (PRR) for the increase in cigarette consumption.

	Comparison Increase vs. No Change	
	PRR (95% CI)	*p*-Value
Age		
≤30 years	Ref.	
31–60 years	1.67 (0.79; 3.53)	0.1807
>60 years	2.06 (0.88; 4.84)	0.0978
Gender		
Female	Ref.	
Male	0.94 (0.45; 1.98)	0.8787
BMI (kg/m^2^)	1.02 (0.95; 1.09)	0.6009
Chronic diseases		
No	Ref.	
Yes	0.49 (0.21; 1.16)	0.1064
Educational level		
No University	Ref.	
University	0.68 (0.40; 1.16)	0.1615
Work situation		
Usual workplace	Ref.	
Unemployed	1.01 (0.43; 2.37)	0.9735
Smart Working	1.00 (0.48; 2.99)	0.9922
Suspension	0.79 (0.32; 1.93)	0.6028
House size		
Less than 100 m^2^	Ref.	
More than 100 m^2^	0.65 (0.39; 1.10)	0.1121
Cohabitation		
alone	Ref.	
not alone	0.61 (0.33; 1.12)	0.1123
Relatives/ himself with COVID-19		
No	Ref.	
Yes	1.11 (0.66; 1.87)	0.6988
Food		
No change	Ref.	
Decreased	0.88 (0.31; 2.48)	0.8111
Increased	1.95 (1.02; 3.73)	0.0433
Sleep quality		
No change	Ref.	
Improved	2.96 (1.30; 6.78)	0.0100
Worsened	2.10 (1.17; 3.76)	0.0124
Physical activity		
No change	Ref.	
Decreased	0.93 (0.43; 2.02)	0.8539
Increased	0.83 (0.31; 2.20)	0.7043

## Supplementary Materials

The following are available online at https://www.mdpi.com/1660-4601/17/17/6287/s1, supplementary materials: questionnaire.
