# Supplementary material for: Determinants of the Lifestyle Changes during COVID-19 Pandemic in the Residents of Northern Italy
Source: Int J Environ Res Public Health. 2020 Aug 28;17(17):6287. doi: 10.3390/ijerph17176287 (PMC7504331; doi:10.3390/ijerph17176287)
Supplement: Supplementary file 1 [file ijerph-17-06287-s001.pdf]

-Questionnaire-

-Indicate your gender: (F/M)

-Enter your age: ______

- Educational level:

Secondary school/no University

University

-Working activity, occupation

Unemployed

Student

Employee (public offices, banks, shops, services)

Healthcare worker (OSS, nurse, doctor)

Business/owner

Freelance

Retired

Housewife

-Indicate how your work situation has changed due to covid-19 restrictions:

It hasn't changed, I go to work

Stopped, I am no longer working

I work from home (smart Working) without going to the office

I'm using a leave (for minors or family assistance)

I'm studying

-Have you had cases of relatives positive at Covid-19?

No

Yes, myself

Yes, a relative of mine

-Indicate the postcode of the area where you live:

____

-Who are you living with at home these days?

Alone

With parents

With partner (wife / husband / partner)

With children and partner

With other relatives

With other cohabitants

-In total how many people? (including yourself):

____

-Indicates the size of the house you are living in

< 100 sqm

> 100 sqm

-Do you have a space outside the house?

Yes, balcony or terrace

Yes, a private garden

Yes, a courtyard

None

-Indicate your weight (in Kg):

____

-Indicate your height (in meters):

____

-Do you suffer from one or more chronic diseases (e.g. diabetes, respiratory diseases, hypertension, motor disabilities, etc.) or long-lasting health problems?

Yes

No

-Do you think you have increased your consumption of daily cigarettes since you were at home?

Yes

No

No, I don't smoke

-How would you define your night sleep during lockdown?

Improved, I sleep more and better

Worsened, I'm suffering from insomnia

It remained as before the lockdown in terms of duration and quality

-How has your sleep changed during lockdown? (multiple answers possible)

I sleep on average 1-2 hours less

I sleep 1-2 hours on average more

I sleep the same hours of sleep as before

I wake up more rested

I wake up less rested

I take an afternoon nap that I didn't do before

-Did you exercise before the pandemic restrictions?

Yes, 4 or more hours per week

Yes, 2-3 hours a week

Yes, on average 1 hour per week

No, I wasn't doing physical activity

-In this pandemic period, compared to before lockdown, you can do physical activity?

Less than usual

Like before

More than before

I don't do any physical activity at home

-Can you quantify how much you are eating during lockdown?

More than usual

Less than usual

Like before

I don't know

-Has your weight changed since the period of limitations due to COVID-19?

I lost 1-2 kg

I increased 1-2 kg

I increased >3 kg

I don't know, I never weigh myself

My weight hasn't changed

-How do you evaluate the quality of your nutrition compared to before isolation for covid-19?

Worst

Like before, Unchanged

Improved

-In this period, have you ever experienced not feeling hungry and skipping meals?

Yes, often

Yes, occasionally

No, never

-What meals do you consume on a regular basis during these isolation weeks?

I don't eat regular meals

breakfast lunch and dinner

breakfast, lunch and dinner and snacks / appetizers

lunch and dinner (I don't have breakfast)

breakfast and lunch and snacks / appetizers (not dinner)

breakfast and dinner (not lunch)

-How many times do you shop foods?

Once a week

Several times a week

Every 15 days

Once a month

-Do you make a shopping list for foods?

No

Yes

-What foods have you bought the most in the last last 15 days)? *(possible to indicate more choices as long as they are more relevant)*

Bread, pasta, flours

Canned food (legumes, tuna, canned meat)

Fresh food (meat, fish, eggs, dairy products)

Fresh fruit and vegetables

Frozen vegetables

Cakes, biscuits or cake ingredients (sugar, eggs, butter, yeast)

Sausages (salami, mortadella, coppa, Wurstel)

Ham/bresaola

Pre-cooked or frozen products (ready meals)

Chips and snacks

Chocolate, ice cream, biscuits and sweet snacks (candies, chewy etc.)

-Which drinks have you bought most in the last period (last 15 days)?

Bottled Water

Fruit juices

Sodas

Beer and wine

Spirits

None of the above

-Have you introduced supplements (vitamins, minerals) to your diet to strengthen your immune system during lockdown?

No

Yes

Not on a regular basis

-Are you practicing food choice precautions that you plan to keep even after the COVID19 emergency ends? *(multiple answers allowed)*

Eat healthier

Eat more fish

Eat more seasonal fruits and vegetables

Eat less meat

Reduce salt

Eat less sugar and sweets

Eat fewer pre-cooked or frozen foods

Drink less alcohol (beer and wine)

Drink fewer spirits

Drink more water

None of the above

-What are the main reasons that guide your food choices during shopping during lockdown? *(maximum 2 answers)*

Difficulty going to supermarkets / food grocery stores

Need to keep costs down

Cooking to spend time

They have not changed

No idea
